# Lingual Abscess in a Psychiatric Patient: A Case Report

**DOI:** 10.1155/2012/194292

**Published:** 2012-01-12

**Authors:** D. Kikidis, K. Marinakis, J. Sengas, A. Chrysovergis

**Affiliations:** ENT Department, University of Athens, Hippokration Hospital, 11527 Athens, Greece

## Abstract

We present a 46-year-old psychiatric patient presenting with a lingual abscess. This paper covers the epidemiology, clinical features, diagnosis, and differential diagnosis with a view to assisting emergency physicians in the timely recognition and management of this rare but potentially life-threatening condition.

## 1. Introduction

Lingual abscess is considered to be a rare condition. It may obstruct the upper airway and become a potentially life-threatening condition. Patients are usually between 30 and 50 years of age [1].

## 2. Case Report

**Figure 1:**
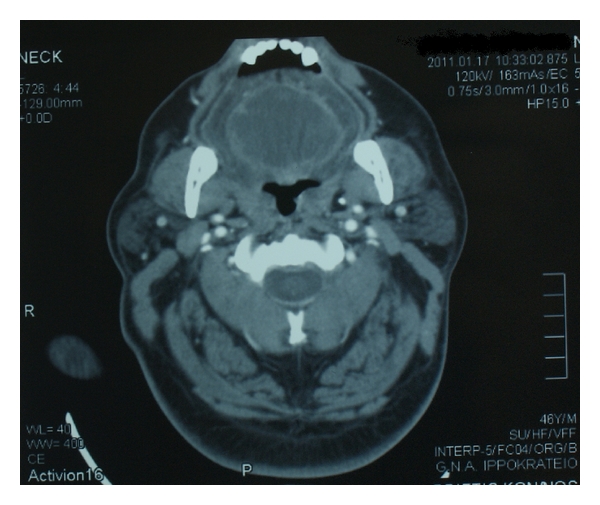
Computed tomographic evidence of a circumscribed mass within the tongue musculature, compatible with a lingual abscess.

A 46-year-old male presented at the ENT emergency department with a seven-day history of painful swelling of the tongue and difficulty in swallowing. The patient was previously seen by a general practitioner who had diagnosed tongue edema and had prescribed oral steroids and antihistamines. The patient was afebrile on presentation, and there was no history of local trauma or recent upper respiratory tract infection. On examination, a swelling of the tongue was apparent, but this did not compromise his airway. He had poor dental health, and the rest of the examination was unremarkable. His medical history included schizophrenia, and he was on Olanzapine. Due to the high suspicion of a possible suppurative process and the patient's limited communication, a CT scan of the head and neck was performed, which showed a circumscribed mass within the tongue musculature, compatible with a lingual abscess (Figure 1).

A needle aspiration of the abscess was performed, followed by incision and drainage. The patient was commenced on amoxicillin and clavulanate potassium and metronidazole to cover both aerobes and anaerobes. No specific microorganism was cultured. Clinical improvement was remarkable with the swelling and pain quickly subsiding. The patient was discharged after a couple of days of hospitalization in complete remission.

## 3. Discussion

Lingual abscess is a rare entity, with only 69 cases reported in the literature. The tongue is considered to be resistant to infectious processes due to its mobility, its thick covering of keratinized mucosa, and its rich vascular supply. These conditions prevent inflammation and make abscess formation rare. Possible risk factors leading to suppuration include trauma, foreign bodies, tongue piercing, and self-injury.

Most of the abscesses are located in the anterior two-thirds of the tongue [2]. Abscesses located in the posterior third are more likely to cause airway obstruction and in these cases, a tracheotomy should be seriously considered at the time of diagnosis.

The pathogens that are usually isolated are streptococci, staphylococci, and anaerobes [3]. Consequently, antibiotics covering this spectrum should be considered as an initial empirical treatment.

The commonest differential diagnosis to consider is angioneurotic edema. This is a vascular reaction occurring both on the skin and in the mucous membrane [4]. In cases of occurrence on the tongue, it can be potentially life-threatening. Symptoms include dysphagia, fullness of the floor of the mouth, and muffle voice, all present in our case. Mortality ranges between 25 to 30%, as a consequence of obstruction of the upper airway [5, 6]. Differential diagnosis of lingual abscess should also include hemorrhage, neoplasia, anaphylaxis, infected dermoid cysts, lingual artery aneurysm, lingual tonsillitis, tuberculosis, actinomycosis, and metabolic macroglossia [7, 8].

Diagnosis of lingual abscess is confirmed by computed tomography, and treatment should include intravenous antibiotics as well as incision and drainage of the abscess. The latter is reported as being a safe and effective treatment in the majority of cases in the literature [3, 8].

Other oral conditions in patients with mental disorders include dry mouth, low saliva rates, halitosis, taste changes, burning syndrome, and bruxism [9]. Statistically significant differences in such disorders between psychiatric patients and healthy controls have been reported. Still it is not clear if some of these lesions and symptoms are related to side effects of mental disorders treatment or the disease itself. Some of these conditions could be risk factors for the occurrence of lingual abscess. We hypothesize that, at least in our case, the lingual abscess was the result of self-injury, which is a behavior commonly observed in patients with mental disorders, such as borderline personality, depression, and schizophrenia [10]. It has already been recognized that patients with schizophrenia appear to be relatively insensitive to physical pain. This special characteristic leads them to receive medical attention relatively late in the physical course of their disease [11–13]. This may be the reason for late presentation in our case as well.

## 4. Conclusion

Lingual abscess is a rare but potentially life-threatening condition. Mental patients may be candidates since they suffer from oral conditions, which are known risk factors for occurrence of lingual abscess. Diagnosis of head and neck abscesses in such cases could be missed due to misleading history because of pain insensitivity.
